# Rethinking dementia in the oldest old: Lessons to learn for the diagnosis and treatment of Alzheimer’s disease

**DOI:** 10.4103/NRR.NRR-D-25-00312

**Published:** 2025-06-19

**Authors:** Chiara Giuseppina Bonomi, Caterina Motta, Martina Gaia Di Donna, Martina Poli, Giacomo Koch, Alessandro Martorana

**Affiliations:** Memory Clinic and Neurodegenerative Dementia Research Unit, Policlinico Tor Vergata, University of Rome “Tor Vergata,” Rome, Italy; Department of Clinical and Behavioral Neurology, Santa Lucia Foundation IRCCS, Rome, Italy; Department of Neuroscience and Rehabilitation, University of Ferrara, and Center for Translational Neurophysiology of Speech and Communication (CTNSC), Italian Institute of Technology (IIT), Ferrara, Italy

Dementia and Alzheimer’s disease (AD) are both age-related conditions that predominantly affect older adults. According to prevalence studies, the burden of these diseases on society is expected to increase in the coming years, particularly in relation to rising longevity and life expectancy. Advances in therapeutic and preventive strategies are needed to help reduce their global burden, which remains among the most significant health challenges in aging populations (Brookmeyer et al., 2007).

In recent decades, scientific efforts have aimed to identify therapeutic strategies that can cause even small delays in the onset and progression of dementia, with special attention to AD. However, randomized clinical trials and research tend to focus on younger elderly populations, typically individuals in their early 70s, creating a disconnect between study cohorts and real-world patients seen in memory clinics. Indeed, while clinicians tend to prioritize helping younger patients as much as possible, as they are more easily referred to clinical trials than older adults, the majority of patients seeking advice for memory deficits tend to be older, partly because younger individuals are less inclined to seek evaluation and partly due to the increasing prevalence of dementia with age.

This discrepancy may raise concerns about the applicability of emerging treatments, particularly in light of the recent approval of disease-modifying anti-amyloid therapies by both the Food and Drug Administration and the European Medicines Agency. These drugs have demonstrated a modest slowing of cognitive decline, which has been regarded as a therapeutic benefit, especially considering the overt failure of previous randomized clinical trials. However, while these advancements represent a turning point in AD management, their implementation in the oldest old remains fraught with challenges.

Individuals over 80 years of age represent the fastest-growing segment of the population affected by dementia and present unique challenges that complicate both diagnosis and treatment. For instance, disease progression in this age group might be slower and potentially influenced by factors such as frailty and cognitive reserve, and accurate neuropsychological assessment remains difficult due to the scarcity of age-specific normative data and the frequent overlap with conditions such as depression and delirium. As of 2025, clinicians are confronted with two major, provocative issues: first, the availability of pharmacological treatments that, at least in theory, could be prescribed to these patients and, second, the emergence of non-invasive diagnostic tools such as plasma biomarkers, which overcome traditional barriers to diagnosis in late life, including concerns about invasiveness and cost-effectiveness. However, despite these breakthroughs, fundamental questions persist regarding patient eligibility for anti-amyloid treatments, the role of coexisting pathologies, and the clinical relevance of diagnosing AD at an advanced age, all of which challenge the practical feasibility of these new approaches in the very elderly.

The first important issue stems from the difficulty of obtaining an accurate biological diagnosis, as older adults are a complex category that does not necessarily fit neatly into standardized diagnostic categories. Specifically, interpreting biomarker analyses to obtain a precise diagnosis is challenging because of the increasing prevalence of age-related AD neuropathological changes (i.e., neurofibrillary tangles and plaques) – that may or may not correlate with clinical symptoms – and of copathologies such as alpha-synuclein or transactive response DNA binding protein of 43 kDa (TDP-43) deposition (Spina et al., 2021). Indeed, traditional single-neuropathology models of dementia become less relevant with aging, as mixed pathology, characterized by the coexistence of multiple neurodegenerative and cerebrovascular pathologies, has emerged as the predominant driver of cognitive impairment in this population. Additionally, vascular comorbidities and other chronic conditions play crucial roles in cognitive decline, often interacting with neurodegenerative processes. Hypertension, diabetes, coronary artery disease, and chronic kidney disease have all been implicated in exacerbating neurodegeneration and could contribute not only to cognitive decline. Moreover, comorbidities in the elderly could also increase the risk of adverse effects from anti-amyloid therapies (Bonomi et al., 2024). Would these therapies be equally effective and safe in patients with mixed pathologies? Should alternative therapeutic targets be explored?

Conversely, a growing body of evidence suggests that not all cases of late-onset dementia are solely attributable to AD pathology. The discovery of Limbic-Predominant Age-Related TDP-43 Encephalopathy (LATE) has further complicated diagnostic and therapeutic strategies. LATE, characterized by TDP-43 protein aggregation in the medial temporal lobe, mimics the amnestic syndrome of AD in approximately 20%–30% of older patients (Montine et al., 2022). Importantly, LATE and AD pathology frequently coexist, challenging the assumption that amyloid deposition is the primary driver of cognitive decline in the oldest population. As individuals age, the likelihood of being diagnosed with AD alone decreases, while the prevalence of LATE rises, highlighting the importance of considering alternative neuropathological processes in cognitive impairment. Autopsy-confirmed cases of LATE often present with an amnestic cognitive disorder that is difficult to distinguish from amnestic mild cognitive impairment due to AD during life, further complicating clinical diagnosis. Adding substantial complexity is the frequent co-occurrence of AD and LATE within the same brain, particularly in overlapping limbic regions, where the presence of both pathologies markedly accelerates cognitive decline. While “pure AD” is known to progress more rapidly than “pure LATE,” individuals with combined AD-LATE pathology experience an even faster rate of cognitive deterioration. The ability to distinguish between these conditions is becoming increasingly relevant with the development of biomarkers that allow for the *in vivo* diagnosis of LATE-neuropathological change (LATE-NC) *versus* ADNC (Wolk et al., 2025).

Indeed, biomarkers not only facilitate early detection but also allow for better differentiation between AD and other neurodegenerative conditions. Their increasing reliability and accuracy have revolutionized diagnostic workflows, making biological definitions of AD more feasible in routine clinical practice. This is particularly important considering amyloid-targeting treatments, which may be less effective in cases where LATE-NC or other neuropathological changes play a more dominant role in neurodegeneration. Using biomarkers offers the potential for more precise treatment strategies, and recent studies have shown that their implementation increases diagnostic certainty in geriatric clinical practice, even in older patients (Decaix et al., 2025). Moreover, while traditional biomarkers such as positron emission tomography tracers and cerebrospinal fluid analyses are often limited by their restricted availability outside specialized healthcare settings, blood-based biomarkers have the potential for widespread clinical implementation and have been formally integrated into the latest revisions of AD diagnostic and staging criteria (Jack et al., 2024). Thus, should biomarker testing be offered to all patients, regardless of age? Should we pursue a more precise diagnosis despite uncertainties regarding the eligibility of older patients for anti-amyloid treatments? In other words, do only those elderly patients who can be treated deserve a diagnosis (e.g., excluding those on anticoagulants, APOE4 carriers, or those with a history of previous hemorrhages)?

Until recently, therapeutic options for cognitive decline did not require a precise biological diagnosis. Without a doubt, a shift from clinical to biomarker-confirmed AD diagnosis in old patients would be driven by the need for more accurate patient selection for anti-amyloid therapy. Arguably, one of the main obstacles to applying AD therapies in the elderly would be the widespread perception among clinicians that their impact on the patient’s condition may be limited compared with other medical interventions. This perception stems from several factors. First, the complexity of older patients, who often present with multimorbidity and frailty, means that dementia is just one of many issues to manage and is not always perceived as the most pressing. In many cases, cognitive decline is seen as a natural part of aging rather than a treatable condition with specific interventions. Additionally, unlike other chronic diseases with well-established treatments that provide clear and immediate benefits, such as hypertension or diabetes management, the effects of anti-amyloid therapies are less tangible in the short term. Their impact is mainly observed in a modest reduction in the rate of cognitive decline rather than in a noticeable improvement in quality of life. Overall, this could tempt clinicians to rely on other more satisfying interventions, with the reassurance of still being able to counteract cognitive decline in a safer way. Additionally, the practical challenges of administering lifelong infusion-based therapies in frail patients with multimorbidity must be considered. This leads many clinicians to believe that the diagnostic and therapeutic efforts may not be justified by the expected benefits, especially given the strict eligibility criteria for these treatments. The belief that only a marginal impact can be achieved in very elderly patients, combined with the risks and high costs of new therapies, contributes to a sense of skepticism and caution in their clinical application.

However, there is the matter of resilience, in that late-onset dementia could indicate a greater resistance to progressive damage or, alternatively, represent a form of “organ failure,” where the brain’s capacity is gradually exhausted. Would these more resilient patients show a better or worse response to amyloid-targeting treatments? Answering this question remains crucial, as it could be key to unravel the potential impact of treatment not only on individual health but also on the broader societal health burden.

**Figure 1:**
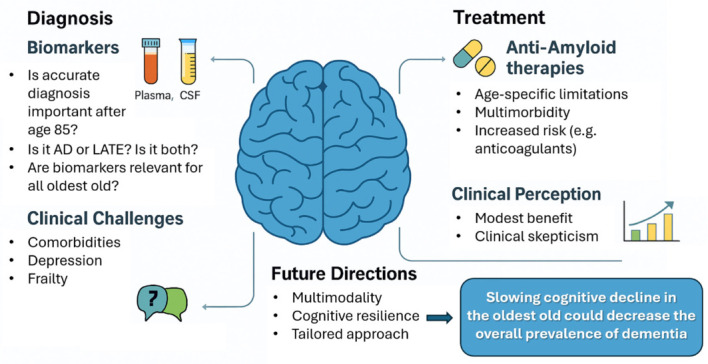
Complex landscape of dementia in the oldest old. AD: Alzheimer’s disease; CSF: cerebrospinal fluid; LATE: Limbic-Predominant Age-Related TDP-43 Encephalopathy.

Slowing cognitive decline precisely in the age group most affected by dementia could be crucial in reducing its overall prevalence and, moving forward, a more personalized and holistic approach to dementia care is needed—one that takes into account not only amyloid pathology but also the broader spectrum of factors contributing to cognitive decline in aging individuals (**Figure 1**). Future research should prioritize the validation of plasma biomarkers, the study of cognitive resilience, and the development of multimodal interventions that address both neurodegenerative and systemic contributors to dementia. Only through such an integrated approach can we hope to improve outcomes for the growing aging population affected by cognitive decline.
